# Identifying Psychosocial Variables That Predict Safer Sex Intentions in Adolescents and Young Adults

**DOI:** 10.3389/fpubh.2016.00074

**Published:** 2016-04-20

**Authors:** Phil Brüll, Robert A. C. Ruiter, Reinout W. Wiers, Gerjo Kok

**Affiliations:** ^1^Department of Work and Social Psychology, Maastricht University, Maastricht, Netherlands; ^2^Department of Developmental Psychology, University of Amsterdam, Amsterdam, Netherlands

**Keywords:** public health, adolescence, sexual risk behavior, adolescent behavior, RAA, sexuality, safer sex motivation, HIV/AIDS

## Abstract

Young people are especially vulnerable to sexually transmitted infections (STIs). The triad of deliberate and effective safer sex behavior encompasses condom use, combined with additional information about a partner’s sexual health, and the kind of sex acts usually performed. To identify psychosocial predictors of young people’s intentions to have safer sex, as related to this triad, we conducted an online study with 211 sexually active participants aged between 18 and 24 years. Predictors [i.e., perceived behavioral control (PBC), subjective norms, and intention] taken from Fishbein and Ajzen’s Reasoned Action Approach (RAA), were combined with more distal variables (e.g., behavioral inhibition, sensation seeking, parental monitoring, and knowledge about STIs). Beyond the highly predictive power of RAA variables, additional variance was explained by the number of instances of unprotected sexual intercourse (SI) during the last 12 months and reasons for using barrier protection during first SI. In particular, past condom non-use behavior moderated PBC related to intended condom use. Further, various distal variables showed significant univariate associations with intentions related to the three behaviors of interest. It may, therefore, be helpful to include measures of past behavior as well as certain additional distal variables in future safer sex programs designed to promote health-sustaining sexual behavior.

## Introduction

Each year, an estimated half a billion new curable sexually transmitted infections (STIs) occur worldwide (1), with young people aged 15–24 years (2) acquiring nearly half of them (3). Reduced access to quality STI prevention services (e.g., due to lack of health insurance, lack of monetary resources, and discomfort with using the available facilities) mean that young people are more prone to both acquiring STIs and to infecting others (3, 4). Another factor which makes young people especially vulnerable to STIs is their often widely divergent views on what exactly defines sexual intercourse (SI) (5, 6). For instance, some young people only define penile–vaginal – but not penile–anal – penetration as SI (7). This finding is particularly alarming in light of the increased risk (as compared with previous estimates) of acquiring human immunodeficiency virus (HIV) *via* receptive anal intercourse (8). In addition, the relatively short average duration of young peoples’ sexual relationships particularly those aged 15–19 years, whose relationships tend to last only a few weeks, adds to the problem (9). To complicate matters even further, young people tend to show a discrepancy between, on the one hand, an increase in reward-seeking behavior and, at the same time, an under-developed capacity for effective self-regulation in risky situations (10–13).

According to WHO data, only about 41% of young people with multiple partners reported using condoms the last time they had sex (14). As Nahmias and Nahmias (15) have pointed out, safer sex behavior is complex. It includes sub-behaviors such as evolved patterns of sexual partnering and promiscuity in increasingly heterogeneous sexual networks that are associated with travel, sometimes on a global scale. Therefore, sub-behaviors – as well as normative factors, availability and costs – need to be addressed in the development of programs designed to promote safer sex.

Encouraging the use of safe sex sub-behaviors could be a promising approach when it comes to the reduction of HIV infection rates. Specifically, the relative risk of HIV infection is reduced 47-fold by choosing a sex partner who has tested negative (as compared to an untested sex partner). Using condoms further reduces the risk 20-fold; and abstinence from penetrative anal sex in favor of penetrative fellatio reduces the risk 13-fold (16, 17).

As laid out in protocols for the design of health promotion programs, such as Intervention Mapping (18), health-related behavior is complex in nature. However, it can be broken down into different levels of comprehensive sub-behaviors that can each be targeted by tailored interventions. Identifying and understanding relevant sub-behaviors – including their determinants at different levels – is a crucial step in promoting health-sustaining behavior.

One explanatory model that can be used to identify determinants of intention to perform safer sex behaviors is the Reasoned Action Approach (RAA) (19). This approach evolved from the Theory of Reasoned Action (TRA) (20) and the Theory of Planned Behavior (TPB) (21, 22). These models of human behavior suggest that behavioral decisions are made based on rational considerations derived from available information. RAA states that behavioral intention – defined as the readiness to engage in a behavior and hence determining the actual behavior – depends on attitude, perceived norms (PN), and perceived behavioral control (PBC) (19). Attitudes are defined as positive or negative evaluations of behavior. PN are defined as perceived social pressure regarding the performance or non-performance of a certain behavior, and PBC is defined as perception of the ease or difficulty related to the performance of a certain behavior. With regard to explaining risky behavior, it has been observed that young people are more likely to engage in riskier behaviors compared to older age groups [e.g., Ref. (23, 24)]. However, it has been convincingly argued that young people do not have flawed reasoning capabilities or poor decision making skills *per se*. Under ideal conditions, they have an almost perfect ability to make rational decisions in order to achieve their goals (25, 26). It is only in the heat of the moment, in unfamiliar situations, in the presence of peers and when behavioral inhibition is required to achieve a health-sustaining outcome that young people tend to reason more poorly than adults (12, 13, 27, 28).

Moreover, according to Montaño and Kasprzyk (29), demographic variables, personality, and individual differences “may be associated with behaviors, but their influence is indirect” (p. 81). To test whether distal variables could be additional predictors of safer sex behavior, this study extended the RAA-guided prediction model by including the following distal variables, in addition to assessing past behavior (i.e., protective measures taken during last SI).

– *Behavioral Inhibition*: Sexual gratification is closely related to human pleasure and strongly influences human behavior (30). Being able to inhibit the promise of pleasure that is potentially health-compromising is an essential component of health-sustaining behavior [e.g., Ref. (31)].
– *Sensation Seeking*: Zuckerman (32) defined sensation seeking as a personality trait characterized by “the need for varied, novel, and complex sensations and experiences, and the willingness to take physical and social risks for the sake of such experiences” (p. 10). Adolescent risk behaviors have consistently been linked to sensation seeking [e.g., Ref. (33)], making this trait a possible predictor of health-compromising sexual behavior.
– *Parental Monitoring*: Parenting has been closely linked to late adolescent (15–19 years) sexual risk behaviors [e.g., Ref. (34, 35)]. However, less is known about the predictive power of parenting as related to intentions to ask a sex partner about his/her health status, perform less risky sex acts, or use condoms during next SI.
– *Knowledge of STIs and Their Transmission*: Knowledge about STIs and knowledge of HIV prevention varies widely within the 15- to 19-year-old age group, and there are no reliable data (including most Western European countries) currently available on this topic (36). However, having knowledge about STIs and their prevention is a key precondition to health-sustaining sexual behavior (14).

Based on the per-act risk of acquiring HIV (8, 16, 17), and on the fact that adolescents often underestimate their risk of acquiring STIs [e.g., Ref. (37, 38)], we included the following specific sub-behaviors, and looked for variables predicting the intention to (1) ask a sex partner about his/her health status, (2) perform less risky sex acts, and (3) use condoms when next having SI with a new partner. Using this framework, we expect that (a) in line with RAA, PN and PBC will predict the intention to perform each aforementioned safer sex sub-behavior and that (b) according to Montaño and Kasprzyk (29), distal, social, and past behavior variables will have indirect effects on the intention to perform each of the three aforementioned safer sex precautions through more proximal RAA variables. Due to a technical problem during data collection, it was not possible to measure attitudes toward target behaviors 1–3 (see Limitations).

## Materials and Methods

### Participants and Procedure

Participants aged between 18 and 24 years who were not in a relationship (where safer sex precautions might not play such a vital role) at the time of the survey were recruited from a German online panel. This panel specializes in social and academic research and offers the option of selecting participants according to their age and demographics (www.meineumfrage.com). After participants gave their written consent, they were linked to a secured website that contained the study materials. An introductory page explained the aim of the study, namely, to find out a possible connection between conscious and unconscious factors in the process of decision making concerning safer sex behavior. Participants were rewarded with panel-owned credit points that could be exchanged for money. Furthermore, if they wished to do so, participants could enter a lottery in order to win one of three additional online shopping credit vouchers (€50/- each). Anonymity was assured as e-mail addresses were not linked to individual data. Completing the questionnaire took approximately 25–30 min. A pretest showed that the survey could be completed within 20-min time. The survey allowed for breaks according to individual needs. Completed questionnaires were anonymously returned to the experimenter’s e-mail inbox in such a way that it was not possible to link the panel issued e-mail to the identity of a participant.

Of the 1114 questionnaires that were accessed online, 242 were completed and returned (21.7%). This high drop-out rate is not unusual for this particular panel because participants are encouraged to visit the sites containing study materials and only then to decide whether to participate or not. Of course, visiting the website out of curiosity is counted as access but is only rewarded with panel-owned credit points after completion of the survey. Thirty-one participants were excluded because they did not answer more than 90% of all required questions. This left a sample of 211 participants for analysis.

### Measures

Demographic characteristics and past sexual experience were assessed by asking participants about sex, age, size of their place of residence (big city: equal or more than 1 million inhabitants, city: between 10,000 and 1 million inhabitants, and rural area: less than 10,000 inhabitants), and composition of household (living with parents/family, alone, and shared apartment). Questions about past sexual behavior (39) assessed sexual identity [e.g., Ref. (40)], age at the time of their first SI, age of their sex partner at the time of their first SI, relationship status with their sex partner at the time of their first SI (i.e., met shortly before, superficial friendship, close friendship, loose relationship, close relationship, and married), barrier protection against STIs during their first SI, number of different sex partners during the last 6 months, number of instances of unprotected SI during the last 12 months, and an estimation of whether they might currently have an STI. Furthermore, participants were asked if they had ever taken a HIV test and whether they had been tested for STIs during the last 12 months.

#### Distal Variables

##### Behavioral Inhibition

Eight items [adapted from Ref. (41, 42)] using a 5-point Likert-Scale ranging from “always” to “never” (e.g., “I plan tasks accurately.” – see Appendix S1 in Supplementary Material for a full list of all items used) were used to measure behavioral inhibition. Cronbach’s alpha, based on the mean inter-item correlation (a Cronbach’s alpha of greater than 0.60 is satisfactory, and a Cronbach’s alpha of greater than 0.80 is good), was α = 0.32. Correlations between single scale items with outcome variables were also low. The scale was adapted from Barratt’s Behavior Inhibition Scale (42) that in its original form contains 30 items. Due to web-related time and space requirements, only eight representative items were selected for this questionnaire. This relatively small number of selected items may have resulted in a lack of scale reliability. Despite Kline (43) arguing that lower Cronbach’s alpha values can be expected for psychological constructs, and Nunnally and Bernstein (44) proposing that values as low as 0.50 are sufficient to identify a scale as reliable, we nevertheless discarded the behavioral inhibition data from our analysis based on these results.

##### Sensation Seeking

Sensation seeking was measured with eight items (45) (e.g., “I like wild parties.” – see Appendix S2 in Supplementary Material for a full list of all items used) using a 5-point Likert-Scale ranging from “completely disagree” to “completely agree”; α = 0.73.

##### Parental Monitoring

How well parents or legal guardians were informed about the social life of participants during their late adolescent years was measured by 10 items [adapted from Ref. (46)] (e.g., “How well did your parents or legal guardians know the parents or legal guardians of your close friends?” or “How well did your parents or legal guardians know about your activities when you were not at home?” – see Appendix S3 in Supplementary Material for a full list of all items used) using a 7-point Likert-Scale ranging from “not at all” to “very well.” Cronbach’s alpha for these measurements was α = 0.82.

##### Knowledge

Participants’ knowledge about STIs and safer sex practices was indexed by means of 15 true-false questions (e.g., “If a 14-year-old teenager has been recently infected with HIV, the first AIDS-related symptoms could appear as late as in his/her mid-twenties.” – see Appendix S4 in Supplementary Material for a full list of all items used). Participants were directed not to guess, and instead to use the “I am not sure” response option when necessary. A correct answer was given +1 point, and an incorrect answer or an “I am not sure” answer was given 0 points. The sum score for knowledge ranged between 0 and 15 points.

#### RAA Variables

The RAA variables were all measured using 7-point Likert scales ranging from 1 = completely agree to 7 = completely disagree. Scores on items that were included to assess the same variable were averaged into one measure in cases where internal consistency was sufficient (Cronbach’s alpha >0.60 or Pearson *r* >0.30 with two items). Items were recorded such that higher scores reflect a stronger presence of the variable concerned.

##### Intention to Ask a Sex Partner about His/Her Health Status (iHS)

One item assessed the participants’ intention to ask his/her sex partner about his/her health status before s/he would next have SI: “I intend to ask my new sex partner about his/her health status before I next have sex with him/her.”

##### Perceived Behavioral Control Related to Asking a Sex Partner about His/Her Health Status (pbcHS)

Two items (“I am confident to ask my new sex partner about his/her health status before I next have sex” and “Whether I inform myself about my new sex partner’s health status before I next have sex with him/her is up to me”) measured PBC related to asking a sex partner about his/her health status (*r* = 0.34).

##### Subjective Norm toward Asking a Sex Partner about His/Her Health Status (pnHS)

The subjective norm related to asking a sex partner about his/her health status was measured by the following two items (*r* = 0.62): “Most people who are important to me think I should ask my new sex partner about his/her health status before I next have sex with him/her” and “Most people like me think I should ask my new sex partner about his/her health status before I next have sex with him/her.”

##### Intention to Perform Less Risky Sex Acts (iSA)

The item “I intend to perform less risky acts when I have sex with a new partner for the next time” assessed the participants’ intention to perform less risky sex acts (e.g., insertive fellatio instead of insertive anal sex) during their next SI.

##### Perceived Behavioral Control Related to Performing Less Risky Sex Acts (pbcSA)

Two items (i.e., “I am confident that I will perform less risky sex acts when I next have sex with a new partner” and “It is up to me whether I perform less risky sex acts when I next have sex with a new partner”) measured PBC related to performing less risky sex acts when next having SI; *r* = 0.37.

##### Subjective Norm toward Performing Less Risky Sex Acts (pnSA)

The subjective norm related to performing less risky sex acts was measured by the following two items (*r* = 0.66): “Most people who are important to me think I should perform less risky sex acts when I next have sex with a new partner” and “Most people like me think I should perform less risky sex acts when I next have sex with a new partner.”

##### Intention to Use Condoms (iCU)

The item “I intend to use condoms when I next have sex with a new partner” assessed the participants’ intention to use condoms when next having SI.

##### Perceived Behavioral Control Related to Using Condoms (pbcCU)

Two items (“I am confident that I will use condoms when I next have sex with a new partner” and “It is up to me whether I use condoms when I next have sex with a new partner”) measured participants’ PBC regarding the use of condom when next having SI with a new partner; *r* = 0.53.

##### Subjective Norm toward Condom Use (pnCU)

The subjective norm related to condom use was measured by the following two items (*r* = 0.61): “Most people who are important to me think I should use condoms when I next have sex with a new partner” and “Most people like me think I should use condoms when I next have sex with a new partner.”

### Statistical Analysis

Data were analyzed using the predictive analytic computer software SPSS, version 22.0 (Statistical Product and Service Solutions, IBM, New York). Following a descriptive analysis (Table 1), the univariate association between all study variables was analyzed with Pearson correlation coefficients (Table 2). Variables that showed significant associations with intention (i.e., asking a partner about his/her health status, performing less risky sex acts, and using condoms the next time they would have SI) were entered into a hierarchical multiple regression to assess both their unique contribution toward the explanation of intention and the total amount of variance in intention explained by the model. To test for indirect effects of distal variables on intention variables (e.g., PBC), we conducted a hierarchical stepwise regression, in which variables were entered from the linear equation on the basis of their ability to improve *R*^2^ at each successive step. Furthermore, moderation analysis was conducted to test whether distal variables (e.g., sensation seeking) influenced the strength of the associations between RAA and intention variables. First, each predictor was centralized, the moderator was entered into the model, and finally the interaction of these two was entered. If the interaction term was significant after controlling for direct effects of predictor and moderator, the moderation was considered significant. Subsequently, simple effect analysis was conducted to determine the influence of distal variables on the associations of RAA variables with intention. Results were considered significant at *p* < 0.05.

**Table 1:** **Past sexual experiences grouped by sex**.

	Male	Female
Age in years of first sexual intercourse	M = 17.6 (SD = 2.64)	M = 16.9 (SD = 2.17)
Age in years of first sex partner	M = 18.8 (SD = 4.05)	M = 19.8 (SD = 3.53)
Relationship to first sex partner		
Met shortly before intercourse	28% (*N* = 32)	14% (*N* = 14)
Already befriended	55% (*N* = 63)	27% (*N* = 26)
Started relationship	33% (*N* = 38)	59% (*N* = 57)
Used barrier protection during first intercourse	67% (*N* = 76)	77% (*N* = 75)
*N* different sex partners (past 6 months)^a^	*N*_max_ = 14 (M = 2.68, SD = 2.80)	*N*_max_ = 10 (M = 2.34, SD = 2.22)
*N* instances unprotected sex (past 12 months)^a^	*N*_max_ = 80 (M = 9.68, SD = 19.12)	*N*_max_ = 80 (M = 5.88, SD = 15.75)
HIV test taken at least once in lifetime	57% (*N* = 65)	49% (*N* = 48)
STI test taken during past 12 months	12% (*N* = 14)	22% (*N* = 22)
Estimation of not having an STI	92% (*N* = 105)	94% (*N* = 91)

*^a^Numbers estimated by participants*.

**Table 2:** **Pearson correlations of univariate associations between all study variables**.

	DV 1	DV 2	DV 3	1	2	3	4	5	6	7	8	9	10	11	12	13	14	15	16	17	18	19	20	21	22	23	24	25
iHS (D1V 1)	–																											
iSP (DIV 2)	0.225^b^	–																										
iCU (DIV 3)	0.226^b^	0.376^b^	–																									
pbcHS (1)	0.803^b^	0.257^b^	0.274^b^	–																								
pnHS (2)	0.762^b^	0.198^b^	0.283^b^	0.716^b^	–																							
pbcSP (3)	0.294^b^	0.734^b^	0.340^b^	0.373^b^	0.269^b^	–																						
pnSP (4)	0.340^b^	0.634^b^	0.287^b^	0.316^b^	0.419^b^	0.682^b^	–																					
pbcCU (5)	0.271^b^	0.352^b^	0.885^b^	0.384^b^	0.314^b^	0.394^b^	0.285^b^	–																				
pnCU (6)	0.300^b^	0.370^b^	0.785^b^	0.353^b^	0.395^b^	0.357^b^	0.316^b^	0.798^b^	–																			
Knowledge (7)	0.135	0.003	0.164^a^	0.234^b^	0.107	0.110	0.074	0.214^b^	0.191^b^	–																		
Age first SI (8)	0.010	0.117	0.191^b^	0.102	0.110	0.128	0.086	0.208^b^	0.154^a^	0.107	–																	
Age partner first SI (9)	0.016	0.101	0.092	0.023	0.083	0.043	0.067	0.089	0.088	−0.051	0.543^b^	_																
Relat. partner first SI (10)	0.142^a^	−0.014	−0.152^a^	0.130	0.074	0.052	−0.027	−0.130	−0.151^a^	0.172^a^	−0.040	−0.211^b^	–															
Protection first SI (11)	−0.102	−0.146^a^	−0.325^b^	−0.173^a^	−0.062	−0.223^b^	−0.102	−351^b^	−0.259^b^	−0.158^a^	−0.176^a^	−0.012	−0.164^a^	–														
Reason prot. first SI (12)	0.020	−0.097	−0.150^a^	−0.100	0.048	−0.210^b^	−0.043	−0.216^b^	−0.139^a^	−0.209^b^	−0.052	0.074	−0.261^b^	0.733^b^	–													
Unprot. SI 12 months (13)	0.141^a^	−0.112	−0.301^b^	0.113	0.090	0.016	−0.007	−0.204^b^	−0.227^b^	0.007	0.069	−0.062	0.122	−0.012	−0.026	–												
N partners SI 6 months (14)	0.020	−0.186^b^	−0.027	−0.050	−0.049	−0.197^b^	−0.125	−0.009	−0.022	−0.058	−362^b^	−0.156^a^	−0.188^b^	0.156^a^	0.203^b^	−0.062	–											
Estimation STI now (15)	−0.033	0.002	−0.124	−0.167^a^	−0.100	0.010	0.026	−0.159^a^	−0.130	−0.162^a^	−0.121	0.118	−0.111	0.042	0.013	0.056	0.228^b^	–										
HIV Test ever (16)	−0.130	0.134	0.021	−0.077	−0.092	0.070	0.039	0.008	−0.001	−0.203^b^	0.047	−0.027	−0.021	−0.109	−0.072	0.013	−0.047	−0.006	–									
STI test last 12 months (17)	−0.147^a^	−0.021	−0.106	−0.113	−0.131	−0.015	0.005	−0.084	−0.099	−0.091	0.037	0.006	−0.042	0.030	−0.064	−0.022	−0.126	−0.012	0.279^b^	–								
Risk seeking (18)	−0.064	−0.142^a^	−0.021	−0.020	−0.072	−0.149^a^	−0.046	−0.013	0.011	0.053	−0.056	−0.049	0.007	0.002	0.019	−0.012	0.099	0.000	−0.078	0.041	–							
Being analytical (19)	−0.177^b^	0.050	−0.009	−0.177^a^	−0.151^a^	−0.021	−0.063	−0.060	−0.060	−0.233^b^	−0.047	−0.034	−0.074	−0.034	−0.014	−0.053	0.133	0.183^b^	0.003	−0.020	0.074	–						
Improvising (20)	0.010	0.010	−0.013	0.068	−0.009	−0.049	0.066	−0.001	0.032	0.016	−0.046	−0.007	−0.001	−0.007	0.073	−0.032	−0.016	−0.080	−0.020	−0.002	0.870^b^	0.047	–					
Being anticipatory (21)	−0.132	0.015	0.070	−0.151^a^	−0.154^a^	−0.081	−0.081	0.040	−0.007	−0.184^b^	−0.059	−0.018	−0.095	0.119	0.119	−0.121	−0.014	0.110	−0.043	0.030	0.244^b^	0.287^b^	0.152^a^	–				
Impulsiveness (22)	−0.014	−0.070	0.042	0.054	0.077	−0.056	−0.097	0.083	−0.006	−0.004	0.060	−0.050	0.102	0.005	0.090	−0.119	−0.045	−0.108	0.075	−0.057	−0.219^b^	−0.114	−0.206^b^	−0.147^a^	–			
Parental knowledge time (23)	0.170^a^	0.081	0.082	0.120	0.137^a^	0.123	0.144^a^	0.045	0.107	0.017	0.127	0.070	0.280^b^	−0.115	−0.076	−0.069	−0.240^b^	− 138^a^	0.129	−0.087	−0.143^a^	−0.043	−0.042	−0.071	0.021	–		
Parental decision power (24)	0.088	0.094	0.146^a^	0.147^a^	0.109	0.130	0.101	0.095	0.140^a^	0.275^b^	0.235^b^	0.137^a^	0.156^a^	−0.080	−0.072	−0.132	−0.187^b^	−0.133	0.013	0.013	−0.198^b^	−0.007	−0.158^a^	−0.121	0.061	0.374^b^	–	
Breaking parental rules (25)	0.004	−0.086	−0.025	−0.016	−0.005	−0.057	−0.106	−0.071	−0.063	−0.044	−0.074	0.032	0.046	0.021	0.047	0.012	0.072	−0.119	−0.069	0.001	0.042	0.040	−0.018	0.056	0.030	0.150^a^	0.104	_

*^a^Correlation is significant at the 0.05 level (two-tailed)*.

*^b^Correlation is significant at the 0.01 level (two-tailed)*.

## Results

### Demographics

The majority of the respondents were males (54%, *n* = 114), with a mean age of 23.5 years. The mean age of female participants was 22.8 years (46%, *n* = 97). Of all participants, 43% (*n* = 91) were living in a big city of 1 million or more habitants, whereas 38% (*n* = 81) were living in a city of between 10,000 and 1 million habitants, and 18% (*n* = 39) were living in rural areas of less than 10,000 habitants. Most participants (73%, *n* = 154) were living in shared apartments, while 17 (8%) were living alone, and 40 (19%) were living with their parents or family. All participants (*n* = 211) classified themselves as heterosexual. Past sexual experiences grouped by sex are reported in Table 1.

### Factorial Analysis

A principal component analysis was conducted for each distal variable. Factor analysis allowed us to identify components with eigenvalues greater than 1.00 that could be grouped together in order to form new factors. All factor structures were obtained by orthogonal rotation. Only factors with eigenvalues equal to or greater than 1.00 were extracted. A new factor apparently related to “being impulsive” explained 28% of the variance (α = 0.58), a second factor related to “being anticipatory” accounted for 18% of variance (α = 0.61), and a third factor related to “being analytical” explained 14.5% of the variance (α = 0.59). Factorial analysis of “sensation seeking” resulted in a new factor related to “risk-seeking” (α = 0.72) that explained 49% of the variance. A second factor seemingly related to “improvising” (α = 0.70) accounted for 14% of variance. Parental monitoring could be disentangled into three new factors. “Parental knowledge about the activities of participants during the time they were unattended” explained 45% of the variance (α = 0.90). A second factor related to “breaking parental rules” accounted for 14% of variance (α = 0.42), and lastly, parental “decision power” accounted for 13% of variance (α = 0.73).

### Univariate Associations

Table 2 presents the Pearson correlations among the study variables with *r* = 0.10–0.23 indicating a small effect, *r* = 0.24–0.36 indicating a moderate effect, and *r* ≥ 0.37 indicating a large effect (47–49).

#### Positive Univariate Associations with Intention

Strong positive associations were found between the RAA variables subjective norm and PBC and intention for each of the three target behaviors (*r*’s > 0.198). Concerning the distal variables, small significant univariate associations with intention to ask a new sex partner about his/her health status were found for the number of instances of unprotected SI during the last 12 months (*r* = 0.141), for the status of relationship with the first sex partner (*r* = 0.142), and for parental knowledge about activities during unsupervised time (*r* = 0.170). No significant association was identified between intention to refrain from risky sex practices and distal variables. Small significant univariate associations with intention to use condoms during first time sex with a new partner were found for parental decision power (*r* = 0.146), for knowledge about STIs (*r* = 0.164), and for the age of first SI (*r* = 0.191). No moderate and large associations were identified between the distal variables and the three intention measures.

#### Negative Univariate Associations with Intention

Small significant univariate associations with intention to ask a new sex partner about his/her health status were found for having being tested for STIs in the past 12 months (*r* = −0.147) and for being analytical (*r* = −0.177). No moderate and large effects were identified. Small significant univariate associations with intention to refrain from risky sex practices were found for risk-seeking (*r* = −0.142), for barrier protection during first SI (*r* = −0.146), and for the number of different sex partners in the past 6 months (*r* = −0.186). No moderate and large effects were identified. Small significant univariate associations with intention to use condoms during first time sex with a new sex partner were found for reasons to protect during the first SI (*r* = −0.150) and for the status of relationship with the first sex partner (*r* = −0.152). Moderate significant univariate associations with the intention of condom use were found for the number of instances of unprotected SI during the last 12 months (*r* = −0.301) and for protection during first SI (*r* = −0.325). No other significant associations were found between the distal variables and the three intention measures.

### Regression Analysis

We used hierarchical multiple regression to explore relationships between significant correlates of intention. The variance inflation factor (VIF) for all variables remained below 3 so that no multicollinearity was detected [a VIF greater than 10 indicates multicollinearity, see Ref. (50, 51)]. Table 3 shows mean, SD, *R*^2^ and *R*^2^ change, and centralized regression coefficients (betas). In relation to intention to ask a sex partner about his/her health status, the RAA predictors PBC and subjective norms accounted for 71.5% of variance (PBC accounted for 64.5% and subjective norms for an additional 7%). Distal predictors added no significant explanation of the variance.

**Table 3:** **Hierarchical multiple regression showing relationships between significant correlates of intention**.

Intention	Predictors	Mean	SD	SE	**β**	*R* square	*R* square change	Sig. *F* change
Asking for health status	Perceived behavioral control	4.65	1.474	1.200	0.803	0.645	0.645	0.000
	Subjective norm	3.77	1.728	1.074	0.384	0.717	0.072	0.000
	Parental knowledge time unattended	24.31	7.966	1.071	0.055	0.720	0.003	0.140
	Relationship with first sex partner	3.57	1.546	1.072	0.034	0.721	0.001	0.384
	Number unprotected SI 12 months	11.59	29.701	1.070	0.049	0.723	0.002	0.194
	STI test in last 12 months	1.89	0.464	1.070	−0.033	0.724	0.001	0.380
	Being analytical	4.71	1.561	1.072	−0.024	0.725	0.001	0.523
Performing less risky sex acts	Perceived behavioral control	4.58	1.521	1.326	0.734	0.538	0.538	0.000
	Subjective norm	3.70	1.772	1.280	0.251	0.572	0.034	0.000
	Number unprotected SI 12 months	11.59	29.701	1.280	−0.046	0.574	0.002	0.322
	Protection during first sex	1.37	0.644	1.283	0.011	0.574	0.000	0.808
	Risk-seeking	2.62	0.950	1.284	−0.044	0.576	0.002	0.343
Using condoms	Perceived behavioral control	5.84	1.573	0.940	0.885	0.783	0.783	0.000
	Subjective norm	5.39	1.721	0.904	0.218	0.800	0.017	0.000
	Age first sexual intercourse	17.33	2.454	0.906	0.010	0.800	0.000	0.759
	Knowledge about STIs	11.65	2.184	0.906	−0.033	0.801	0.001	0.303
	Parental decision power	10.03	2.992	0.901	0.061	0.805	0.003	0.065
	Protection during first sex	1.37	0.644	0.902	−0.024	0.805	0.000	0.478
	Number unprotected SI 12 months	11.59	29.701	0.878	−0.109	0.816	0.011	0.001
	Relationship with first sex partner	3.57	1.546	0.879	−0.026	0.817	0.001	0.415
	Reasons for protection first sex	3.02	1.527	0.872	0.096	0.820	0.004	0.036

Regarding the intention to perform less risky sex acts, RAA predictors PBC and subjective norms accounted for 57.2% of variance (PBC accounted for 53.8% and subjective norm accounted for an additional 3.4%). Distal predictors added no significant explanation of the variance.

Concerning the intention to use condoms, RAA predictors PBC and subjective norms accounted for 80% of variance (PBC for 78.3% and subjective norms for an additional 1.7%). The number of instances of unprotected SI during the last 12 months explained an additional 1.1% of variance. This was followed by reasons to use barrier protection during first SI, which explained a further 0.5% of variance.

### Moderation Analysis

Next, we conducted a moderation analysis to test if distal variables moderated the relationship between RAA predictor variables and intentions. Only regarding the intention to use condoms, results showed a significant interaction effect between the number of instances of unprotected SI during the last 12 months and PBC toward using condoms (*F* change = 411.07, *B* = 0.004, SE = 0.002, β = 0.082, and *p* = 0.017).

**Figure 1:**
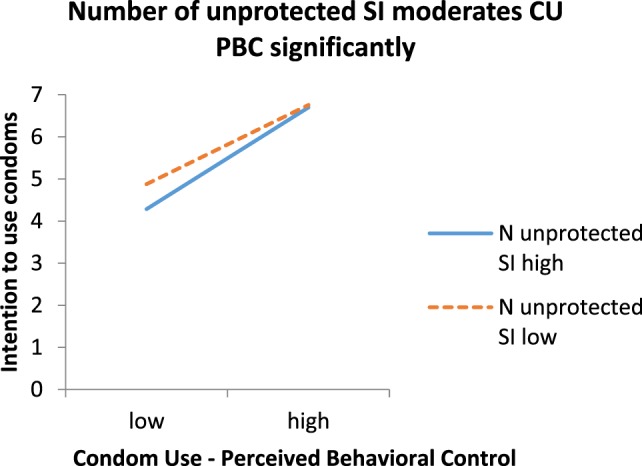
**Regression slopes arising from the relation between perceived behavioral control toward condom use and intention to use condoms with a new sex partner for participants who are relatively high [1 SD (SD = 1.57) greater than the mean, see Ref**. (52)**] versus low (1 SD less than the mean) in the number of instances of unprotected sexual intercourse in the past**.

Simple slope analyses (52) for the association between PBC to use condoms and actual condom use intention were tested for a low (−1 SD below the mean) and a high (+1 SD above the mean) number of instances of unprotected SI during the last 12 months. Each of the simple slope tests revealed a significant association between PBC to use condoms and intention to use condoms, but PBC was more strongly related to condom use intention for a high number of past instances of unprotected SI (*B* = 1.20, SE = 0.06, β = 0.94, and *p* < 0.001) than for a low number (*B* = 0.98, SE = 0.06, β = 0.76, and *p* < 0.001). Figure 1 plots the simple slopes for the interaction.

## Discussion

Perceived behavioral control and subjective norms predicted participants’ intention to perform safer sexual behavior – by up to 80% in relation to condom use. Both of these RAA variables also predicted intention to ask a partner about his/her health status (accounting for 71.4% of variance), as well as intention to perform less risky sex acts (57.2%). Although these values may appear high, according to Fishbein and Ajzen (19), such percentages are not uncommon if, as in this study, the correspondence principle is strictly followed by taking into account factors such as time, action, target, and context. This primacy of RAA variables over distal variables is further accentuated by the fact that analyses of attitude measures could not be included due to a technical problem during data acquisition. RAA variables emerged as the most prominent predictors of intentions related to the safer sex behaviors investigated in this study. The intention to perform less risky sex acts and to ask a new sex partner about his/her health status was low compared to the intention to use condoms. Only 13.3% of all participants reported that they intended to perform less risky sex acts, and only 15.2% intended to ask a new sex partner about his/her health status, whereas 56.9% intended to use condoms the next time they would have sex with a new partner.

### Intention to Ask a New Sex Partner about His/Her Health Status

Perceived behavioral control (64.5%) and subjective norm (7.2%) were the main predictors of intention to ask a new sex partner about his/her health status. However, intention was low. In fact, even if scores of participants who strongly intended to ask a new sex partner about his/her health status were combined (points 6 and 7 of the 7-point Likert-scale measuring intention), only a total of 23.3% of participants intended to perform this behavior. Even if distal variables did not predict intention, parental monitoring during adolescence showed a significantly though small positive univariate association with both intentions to ask a new sex partner about his/her health status, and to use condoms during their first SI with a new partner. This finding is in line with Bronfenbrenner (53) model that accords parents a direct influence on their child. Furthermore, an authoritative parenting style with balanced support and monitoring (54) has been identified as one which promotes optimal child outcomes [e.g., Ref. (55, 56)]. Monitoring not only restricts adolescents’ opportunities to engage in risky behavior (35) but is also a factor associated both with caring about safer sex practices by asking about the health status of a new sex partner and with condom use.

### Intention to Perform Less Risky Sex Acts with a New Partner

Perceived behavioral control (53.8%) and subjective norm (3.4%) were the strongest predictors of intention to perform less risky sex acts. Here too, intention was low. If scores of participants who strongly intended to perform less risky sex acts were combined (points 6 and 7 of the 7-point Likert-scale measuring intention), only a total of 22.3% of participants intended to refrain from risky sex acts with a new partner. One possible reason for this could be a lack of knowledge about the per-act risk associated with various sex acts. Most safer sex programs focus on promoting condom use without emphasizing the risks of certain sex acts (e.g., penetrative anal sex and receptive oral sex). Furthermore, sometimes aberrant definitions of SI [as reported by Mehta et al. (7)] can lead to the performance of high risk sex acts without identifying these as such and hence without feeling a need to restrain from them. However, engaging in risky sex practices despite their possible health threats is in accordance with dual-process theory [e.g., Ref. (11–13)], which states that immediate reward-seeking behavior goes hand-in-hand with higher risk taking and thus with the possibility of risky SI. Interestingly, the percentage of participants intending to perform less risky sex acts is comparable to the percentage of participants willing to ask for a new partner’s health status. Apparently, refraining from risky sex acts and asking about a new partner’s health status were not considered to be rewarding safer sex practices. It is possible that immediate reward seeking in aroused conditions – and in the absence of a matured self-regulatory capacity – could account for not performing these behaviors, as proposed by dual-process theory. Therefore, immediate pleasure gained from riskier sex acts constitutes a considerable risk in terms of acquiring an STI. Consequently, the per-act risk associated with risky sex acts should be emphasized in safer sex programs in order to enhance the intention to perform less risky sex acts with a new partner.

### Intention to Use Condoms with a New Partner

Perceived behavioral control had the greatest influence on intention to use condoms (this variable predicted 78.3% of variance), while subjective norms accounted for a much smaller 1.7% of variance. When measures of participants who strongly intended to use condoms when they next have SI were combined (points 6 and 7 of the 7-point Likert-scale measuring intention, with 7 indicating “completely agree”), a total of 69.7% of participants intended to use condoms. The number of instances of unprotected SI during the last 12 months added 1.1% predictive power and was identified as a moderating variable of PBC related to intention to use condoms. Low PBC toward condom use resulted in only moderate intentions to use condoms with a new sex partner, particularly if the number of instances of previous unprotected SI was high. In their meta-analysis, McEachan et al. (57) showed that if RAA variables were controlled for, past behavior still had a strong effect on intentions. The moderating impact of past behavior on future behavior could be explained by various automatic processes such as those proposed by Strack and Deutsch (58) Reflective Impulse Model, which also takes habit formation into account. Consistent non-use of condoms in the past, hand-in-hand with low PBC related to condom use could in fact lead to the non-use of condoms becoming a habit. This is further underscored by the predictive power of reasons for using barrier protection during first SI on condom use intention. Even if this variable only predicted 0.4% of variance, it shows that reasons for past condom use behavior influence current condom use intention. Such combinations of RAA and distal variables should be taken into account when designing behavior change interventions that focus on strengthening perceived control while at the same time mimicking the effects of successful previous condom use.

In alignment with earlier studies [e.g., Ref. (59–62)], the results indicate the importance of strengthening PBC related to using condoms in sexual education programs in order to increase intention to actually use condoms when next having SI with a new partner. However, PBC regarding condom use is a multidimensional construct (63), and it has been shown that intended and performed adolescent condom use is still inconsistent (64). Increasing our understanding of these intricate interplays between RAA and distal variables could, therefore, allow for a better tailoring of interventions toward an adolescent population. For instance, understanding how past condom non-use as a distal variable moderates the RAA variable PBC, and in this way regulates future action, could help in the design of interventions that simulate successful past condom use behavior among non-users in order to eventually increase PBC related to condom use.

Intentions to perform three safer sex behaviors (performing less risky sex acts, asking a new sex partner about his/her health status, and using condoms) were mainly predicted by RAA variables, namely, PBC and subjective norms. High intention related to condom use was sharply contrasted by a low intention related to asking a new sex partner about current health issues and by a low intention to refrain from sexual practices that have been identified as possibly harmful. Including additional distal variables in safer sex programs may be one helpful way to further promote health-sustaining sexual behavior. Variables such as the level of risk-seeking behavior (concerning the intention to refrain from risky sex acts) or parenting style and unprotected sexual activity (concerning the intention to ask for the current health status) showed significant univariate associations with those intentions. The amount of unprotected SI during the last 12 months did not only add predictive power to the intention to use condoms but also moderated the impact of PBC on intended condom use. Nevertheless, more research is needed to better understand the relationship between distal variables acting as predictors and/or moderators of a specific intention.

### Limitation

Unfortunately, due to a technical problem during data collection, data regarding attitudes concerning the three behaviors of interest could not be collected. Attitudes are important predictors of intention to perform a behavior. However, subjective norms and PBC have also been identified as influential predictors and, as such, should be taken into account when designing safer sex interventions (19). In line with Bandura (65), who emphasized the predictive utility of self-efficacy beliefs on intentions, self-efficacy – a concept closely related to PBC (19) – in particular has been found to improve the predictive power of intentions – more so than attitude and social norms (22). This has been confirmed by several studies that have investigated condom use behavior [e.g., Ref. (66–68)]. In summary, although the interpretation of our data remains hampered by the loss of attitude measurements, our results still illustrate the relative importance of PBC related to condom use behavior in conjunction with social norms and several distal variables.

## Author Contributions

PB: planning, design, execution, data analysis, and report. RR: design, data analysis, and report. RW and GK: report.

## Conflict of Interest Statement

The authors declare no conflict of interest involving the preparation and submission of this manuscript.
