# Supplementary material for: Identifying Psychosocial Variables That Predict Safer Sex Intentions in Adolescents and Young Adults
Source: Front Public Health. 2016 Apr 20;4:74. doi: 10.3389/fpubh.2016.00074 (PMC4837163; doi:10.3389/fpubh.2016.00074)
Supplement: Supplementary file 1 [file table_1.docx]

**Identifying psychosocial variables that predict safer-sex intentions in adolescents and young adults**

**Appendix S1**

**Barratt Behavior Inhibition Scale (adapted from Patton et al., 1995)**

I plan tasks carefully. *[always (1) – never (5)]*

I say things without thinking. *[always (1) – never (5)]*

I buy things on impulse. *[always (1) – never (5)]*

I like to think about complex problems. *[always (1) – never (5)]*

I am self-controlled. *[always (1) – never (5)]*

I change hobbies. *[always (1) – never (5)]*

I save regularly. *[always (1) – never (5)]*

I am future oriented. *[always (1) – never (5)]*

**Appendix S2**

**Brief Sensation Seeking Scale (Hoyle et al., 2002)**

*Experience seeking*

I would like to explore strange places. *[completely disagree (1) - completely agree (5)]*

I would like to take off on a trip with no pre-planned routes or *timetables [completely disagree (1) - completely agree (5)]*

*Boredom susceptibility*

I get restless when I spend too much time at home. *[completely disagree (1) - completely agree (5)]*

I prefer friends who are excitingly unpredictable. *[completely disagree (1) - completely agree (5)]*

*Thrill and adventure seeking*

I like to do frightening things. *[completely disagree (1) - completely agree (5)]*

I would like to try bungee jumping. *[completely disagree (1) - completely agree (5)]*

*Disinhibition*

I like wild parties. *[completely disagree (1) - completely agree (5)]*

I would love to have new and exciting experiences, even if they are illegal. *[completely disagree (1) - completely agree (5)]*

**Appendix S3**

**Parental monitoring (adapted from Beyers & Goossens, 1999)**

How well did your parents or legal guardians know your close friends? *[not at all (1) – very well (5)]*

How well did your parents or legal guardians know the parents or legal guardians of your close friends? *[not at all (1) – very well (5)]*

How well did your parents or legal guardians know where you were when you were not at home? *[not at all (1) – very well (5)]*

How well did your parents or legal guardians know who you met up with when you were not at home? *[not at all (1) – very well (5)]*

How well did your parents or legal guardians know what you did when you were not at home? *[not at all (1) – very well (5)]*

How well did your parents or legal guardians know what you did when they were not at home? *[not at all (1) – very well (5)]*

Please think now about your parents or legal guardians with whom you lived. Who decided at what time you needed to be back home when you went out at night? *[me alone – my parents or legal guardians – me together with my parents or legal guardians]*

Please think now about your parents or legal guardians with whom you lived. Who decided with whom you were allowed to have contact with? *[me alone – my parents or legal guardians – me together with my parents or legal guardians]*

How often did you break the rules about the time you needed to be back home*? [never (1) – always (5)]*

How often did you break the rules about with whom you were allowed to have contact with? *[never (1) – always (5)]*

**Appendix S4**

**Knowledge**

HIV can be transmitted through hugging, kissing, or holding hands. *[yes – no – not sure]*

Someone can transmit venereal diseases without showing symptoms of them. *[yes – no – not sure]*

If someone has had a sexually transmitted disease, s/he can’t get the same disease a second time. *[yes – no – not sure]*

All venereal diseases with the exception of AIDS can be treated and are harmless. *[yes – no – not sure]*

It is possible to have several sexually transmitted diseases at the same time. *[yes – no – not sure]*

Most venereal diseases can be treated without medical treatment. *[yes – no – not sure]*

If symptoms of a sexually transmitted disease are gone the disease is cured. *[yes – no – not sure]*

The breakout of a sexually transmitted disease can be prevented by an intensive washing of genitalia. *[yes – no – not sure]*

A HIV infection could possibly be prevented by a “post exposition prophylaxes” (PEP). *[yes – no – not sure]*

With condoms, water-based lubricants are most suitable. *[yes – no – not sure]*

Condoms could be involuntarily ripped or come off during sexual intercourse. *[yes – no – not sure]*

HIV is caused by AIDS. *[yes – no – not sure]*

AIDS triggers an immune deficiency. *[yes – no – not sure]*

AIDS can be cured. *[yes – no – not sure]*

If a 14 years old teenager has been recently infected with HIV, the first AIDS related symptoms could appear as late as in his/her mid-twenties. *[yes – no – not sure]*
